# Effect of Short-Term Stimulation with Interleukin-1*β* and Differentiation Medium on Human Mesenchymal Stromal Cell Paracrine Activity in Coculture with Osteoblasts

**DOI:** 10.1155/2015/714230

**Published:** 2015-12-22

**Authors:** Jan O. Voss, Claudia Loebel, Jennifer J. Bara, Marc Anton Fussinger, Fabian Duttenhoefer, Mauro Alini, Martin J. Stoddart

**Affiliations:** ^1^AO Research Institute Davos, 7270 Davos Platz, Switzerland; ^2^Department of Oral and Maxillofacial Surgery/Clinical Navigation, Charité-Universitätsmedizin Berlin, Campus Virchow-Clinic, 13353 Berlin, Germany; ^3^Department of Oral and Maxillofacial Surgery, Medical Center-University of Freiburg, 79106 Freiburg, Germany

## Abstract

*Introduction.* Human mesenchymal stromal cells (hMSCs) exhibit the potential to accelerate bone healing by enhanced osteogenic differentiation. Interleukin-1*β* is highly expressed during fracture healing and has been demonstrated to exert a significant impact on the differentiation behaviour of hMSCs. Here, we investigate the effect of 2-hour IL-1*β* stimulation on the differentiation and paracrine activity of hMSCs in coculture with osteosarcoma cells* in vitro*.* Methods.* hMSCs from 3 donors were incubated for 2 hours with 10 ng/mL IL-1*β* and subsequently cocultured with MG63-GFP cells either in control or in differentiation medium in a transwell system for 28 days. Genetic and functional effects were investigated.* Results.* hMSCs cultured in control medium exhibited a regulatory effect on cocultured MG63-GFP cells, resulting in upregulation of osteogenic gene expression in combination with increased ALP activity. However, while stimulated hMSCs cultured under differentiation conditions exhibit signs of osteogenic differentiation, osteogenic differentiation also caused an impaired regulatory effect on the cocultured MG63-GFP cells.* Conclusion.* Short stimulation of hMSCs has the potential to modify their long-term behaviour. In addition, undifferentiated hMSCs are able to regulate osteoblast differentiation; however, this regulatory function is lost upon osteogenic differentiation* in vitro*. This offers a novel approach for clinical cell therapy protocols.

## 1. Introduction

Effective treatment of bone defects attributed to atrophy, trauma, malformation, and radical surgical resection represents a major clinical challenge. Autologous bone graft transplantation is on the one hand beneficial due to lack of induced immune response. On the other hand, it creates the need for secondary surgical procedures, which can cause severe side effects and donor site morbidity [1, 2]. Different cell types are currently being investigated for their potential use in bone repair [3]. In this context, human mesenchymal stromal cells (hMSCs) are rapidly gaining interest for regenerative medicine approaches. As nonhematopoietic stromal cells, hMSCs are capable of self-renewal and exhibit the ability to differentiate along multiple mesenchymal lineages such as osteogenic, chondrogenic, and adipogenic lineages [4]. Besides their direct differentiation potential, transplanted cells may exert a great influence on the surrounding cell microenvironment indirectly by the paracrine secretion of a broad spectrum of cytokines and growth factors [5].

Bone healing in response to fractures is well orchestrated and involves a broad spectrum of cytokines during the different healing phases [6]. Such growth and differentiation factors exert significant impact on resident cells thereby influencing subsequent repair. The importance of this cytokine milieu was demonstrated directly in different* in vivo* approaches [7–9] and indirectly by the delayed fracture healing when hematoma was removed [7]. Proinflammatory cytokines, such as interleukin-1 (IL-1), interleukin-6 (IL-6), and tumor necrosis factor-alpha (TNF-*α*), as well as other inflammatory cytokinesplay an essential role in the initiation process of the repair cascade by mediating inflammation, degradation, and matrix synthesis [10, 11]. The effect of inflammatory cytokines on cells of mesenchymal origin has been investigated in various* in vitro* settings [12–15]. IL-1*β*, as one of the most powerful proinflammatory cytokines, is upregulated in the early phase of fracture healing, presenting bimodal upregulation [10, 16–18]. We and others have described the modulatory effect of long-term IL-1*β* stimulation on the osteogenic differentiation behaviour of hMSCs, however with conflicting results [19–22]. While the exact reasons for this are unclear, differences in study design (young versus older donors, 7-day versus 28-day stimulation) make direct comparison difficult.

Recently, it has been described that short stimulation of human adipose-derived mesenchymal stem cells can influence their differentiation behaviour [23]. In addition, 2-hour short-term stimulation of bone marrow derived hMSCs with IL-1*β* had a strong impact on protein synthesis and influenced the secretion of proteins involved in chemotaxis, inflammation, angiogenesis, and bone formation [24].

The intraoperative usage of autologous hMSCs may be beneficial for the patient as it avoids* in vitro* cell manipulation, which is associated with changes in cell behaviour [25], reduces the risk of contamination, is immunologically compatible, and minimizes surgical intervention to one procedure [26].

Considering the potential future use of transient intraoperative stimulation for therapeutic purposes, we investigated the effect of 2-hour IL-1*β* stimulation on hMSCs as a proof of principle. In order to study the effect of IL-1*β* on hMSCs, we used high density micromass culture, which mimics more closely the cell microenvironment* in vivo* [27]. Additionally, it has been shown that micromasses promote the differentiation of osteoblast-like cells in comparison to monolayer culture [28, 29].

In addition to the direct effect of IL-1*β* stimulation on MSC osteogenic differentiation, their regulatory behaviour towards surrounding cells by indirect coculture was also investigated. Here, we took advantage of the homogenous cell population of the MG63 cell line, an osteosarcoma cell line that exhibits an immature phenotype, arrested at preosteoblastic state, which ensured consistent cell response and conditions when comparing the cell response of different MSC donors [30]. Indirect coculture with MG63 cells constitutively expressing green fluorescent protein (MG63-GFP) was carried out by separating both cells types via a transwell system, which allows the exchange of soluble factors but avoids direct cell-cell contact [31].

## 2. Materials and Methods

### 2.1. Bone Marrow

Human bone marrow was harvested from 3 patients (male : female ratio 1 : 2; mean age 38,33 years; range 25–50 years) with full ethical approval (KEK Bern 126/03) and informed patient consent.

### 2.2. MSC Isolation and Cell Culture

Mononuclear cells (MNCs) were isolated by density centrifugation using Histopaque-1077 (Sigma-Aldrich, St. Louis, USA) as previously described [4]. In brief, the interphase containing the mononuclear cell fraction of whole marrow aspirate was collected. To gate out red blood cells, a cell count above 8 *μ*m was performed using a cell Scepter 2.0 Automated Cell Counter (Millipore, Darmstadt, Germany). Cells were plated as P0 generation at a cell seeding density of 5 × 10^4^/cm^2^ in polystyrene cell culture flasks (TPP, Trasadingen, Switzerland) with *α*-Minimum Essential Medium (*α*MEM, Gibco/Life Technologies, Carlsbad, USA) supplemented with 10% MSC qualified foetal bovine serum (FBS) (Sera Plus, PAN-Biotech GmbH, Aidenbach, Germany), 1% Pen/Strep (10.000 units/mL Penicillin, 10.000 *μ*g/mL Streptomycin; Life Technologies, Carlsbad, USA), and 5 ng/mL basic fibroblast growth factor-2 (bFGF-2, Fitzgerald Industries, Acton, USA). Cells were incubated in humidified atmosphere at 37°C with 95% O_2_ and 5% CO_2_. The first medium change was carried out on day 4 in order to let MSCs attach, after which medium was replaced three times per week. The adherent cell fraction was plated at 3 × 10^3^ cells/cm^2^, grown to confluence, and then cryopreserved in CM (see below) and 10% dimethyl sulfoxide (Sigma-Aldrich, St. Louis, USA) at 2 × 10^6^ cells/mL (stated as P0 generation). Prior to experiments, cells were thawed, seeded at a density of 3 × 10^3^ cells/cm^2^ in culture flasks, and expanded until P2.

### 2.3. Differentiation, IL-1*β* Stimulation, and Micromass Formation

MG63-GFP cell micromass was formed by plating 10 × 10^5^ MG63 cells retrovirally transduced to express green fluorescent protein (MG63-GFP) [32] in 5 *μ*L of control medium (CM containing low glucose DMEM supplemented with 10% FBS and 1% Pen/Strep) on the bottom of a 24-well plate. MG63-GFP have previously been shown to be phenotypically indistinguishable from the parent MG63 population [32]. In order to achieve stable micromass formation, cells were incubated for 3 hours before adding the assigned medium.

HMSCs were incubated as a cell suspension in 3 mL CM for 2 hours with or without 10 ng/mL recombinant human IL-1*β* (PromoKine, Heidelberg, Germany) at room temperature. Cell suspension was shaken every 15 minutes to allow even dispersion. Following a centrifugation step, the medium was discarded and micromasses were formed on 24-well culture inserts (BD Bioscience, Franklin Lakes, USA, 0.4 *μ*m pore size). After 3 h incubation, either CM or osteogenic differentiation medium (DM = CM supplemented with 50 *μ*g/mL ascorbic acid, 5 mM *β*-glycerophosphate, and 10 nM dexamethasone) was added. 24-well inserts were transferred into 24-well plates in order to achieve a micromass coculture system. Medium changes were performed three times per week.

### 2.4. RNA Extraction and Reverse Transcription

RNA extraction of samples on days 0, 2, and 7 and 0, 14, and 21 was carried out by using TriReagent and RNeasy Kit (Quiagen, Hilden, Germany). After adding TriReagent, polyacryl carrier, and 1-bromo-3-chloropropane, the following centrifugation induced phase fractionation. The upper phase containing the RNA was collected, mixed with 70% ethanol, and applied to RNeasy columns. 100 ng RNA was used for reverse transcription (RT). RT was carried out using TaqMan Reverse Transcription kit (Applied Biosystems, Foster City, USA).

### 2.5. Real-Time Polymerase Chain Reaction (rt-PCR)

rt-PCR was performed using QuantStudio 6 and 7 Flex Real-Time PCR System (Life Technologies, Carlsbad, USA). The Ct value of each sample was normalized to the expression of the housekeeping gene RPLP0 (dCt). The expression level of each target gene was calculated as 2^−ddCt^ normalized to dCt values of the sample harvested on day 0 [33]. Primer and probes sequences used are listed in Supplemental Table 1 in Supplementary Material available online at http://dx.doi.org/10.1155/2015/714230.

### 2.6. Deoxyribonucleic Acid (DNA) Assay

On days 14, 21, and 28, micromasses were digested in 0.5 mg/mL recombinant Proteinase K (Roche, Basel, Switzerland) for 16 hours at 56°C. DNA content was measured using Hoechst 33258 (Polysciences, Warrington, USA) assays as described previously [34]. A DNA standard curve was generated using calf thymus DNA (Life Technologies, Carlsbad, USA).

### 2.7. Alkaline Phosphatase (ALP) Protein Activity

Quantitative, colorimetric determination of ALP protein activity was performed by using Kit number 104 (Sigma-Aldrich, St. Louis, USA) according to the manufacturer's instructions. On days 14 and 21, samples were incubated at 4°C for 1 hour in 0.1% Triton-X in 10 mM Tris-HCl. By measuring the absorbance of the ALP product (*p*-nitrophenol) at *λ* = 405 nm, the ALP activity was determined. ALP activity was calculated according to an ALP standard curve. Results are expressed in nmol of* p*-nitrophenol formed per minute related to total DNA amount ((nmol pNP/min) *μ*g DNA^−1^) of parallel cultures.

### 2.8. Calcium-45 (^45^Ca) Radioisotope Incorporation

On day 21, micromass cultures were incubated for 16 hours with [^45^Ca]Cl_2_ (1.25 *μ*Ci/mL) radioisotope (Perkin Elmer, Waltham, USA). Following incubation, the medium was discarded and the samples were washed with PBS. Samples were dissolved in 70% formic acid at 65°C for 1 hour and radioactivity was measured by scintillation counting. Results are expressed in counts per minute (CPM) related to *μ*g DNA (CPM/*μ*g DNA) of parallel cultures.

### 2.9. Alizarin Red S Staining and Quantification

Following the fixation of cells with 4% formaldehyde on day 28, staining was carried out using 40 mM Alizarin Red S (ARS) (Sigma-Aldrich, St. Louis, USA). Quantification was performed by dissolving bound ARS in 10% acetic acid (Sigma-Aldrich, St. Louis, USA). Extracted ARS was detected by measuring the absorbance at *λ* = 405 nm. Results are expressed in nmol of bounded ARS related to *μ*g DNA (nmol/*μ*g DNA) of parallel cultures.

### 2.10. Statistical Analysis

Each experiment was performed in triplicate with 3 different donors. Each donor was tested separately and the data was collated for statistical analysis. Values are presented as mean ± standard error of the mean (SEM). Data was tested for normal distribution by D'Agostino-Pearson omnibus normality test. To test for differences between the groups, one-way analysis of variance (one-way ANOVA) was used followed by Bonferroni's Multiple Comparison* Post Hoc* Analysis according to the normality test result using GraphPad Prism (GraphPad Software, Inc., La Jolla, USA). Statistical significance was considered with *p* < 0.05. ( ^*∗*^ *p* ≤ 0.05,  ^*∗∗*^ *p* ≤ 0.01, and  ^*∗∗∗*^ *p* ≤ 0.001).

## 3. Results

### 3.1. IL-1*β* Stimulation Induces Transient Osteogenesis-Related Gene Expression Changes in Human Mesenchymal Stem Cells

**Figure 1:**
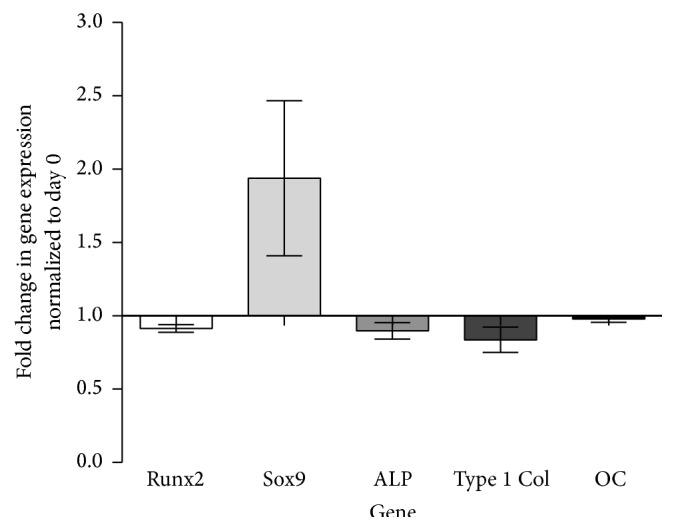
hMSC gene expression profile after 2-hour IL-1*β* stimulation. Fold change in relative gene expression of stimulated hMSCs normalized to unstimulated hMSCs (RPLP at day 0) quantified by real-time PCR. Bars show the mean ± SEM of 6 different experiments performed in triplicate per group (*n* = 18 per group).

In order to investigate the direct effect of IL-1*β* on hMSCs, the gene expression following 2-hour stimulation was analysed. Interestingly, shortly after the stimulation with IL-1*β*, stimulated hMSCs show downregulation of Runx2, alkaline phosphatase (ALP), and Type 1 Collagen (Type 1 Col) compared to unstimulated hMSCs (Figure 1). While these osteogenic related genes decreased, the gene expression of Sox9 (a transcription factor related to chondrogenesis and endochondral bone formation [35]) was strongly upregulated immediately after stimulation.

**Figure 2:**
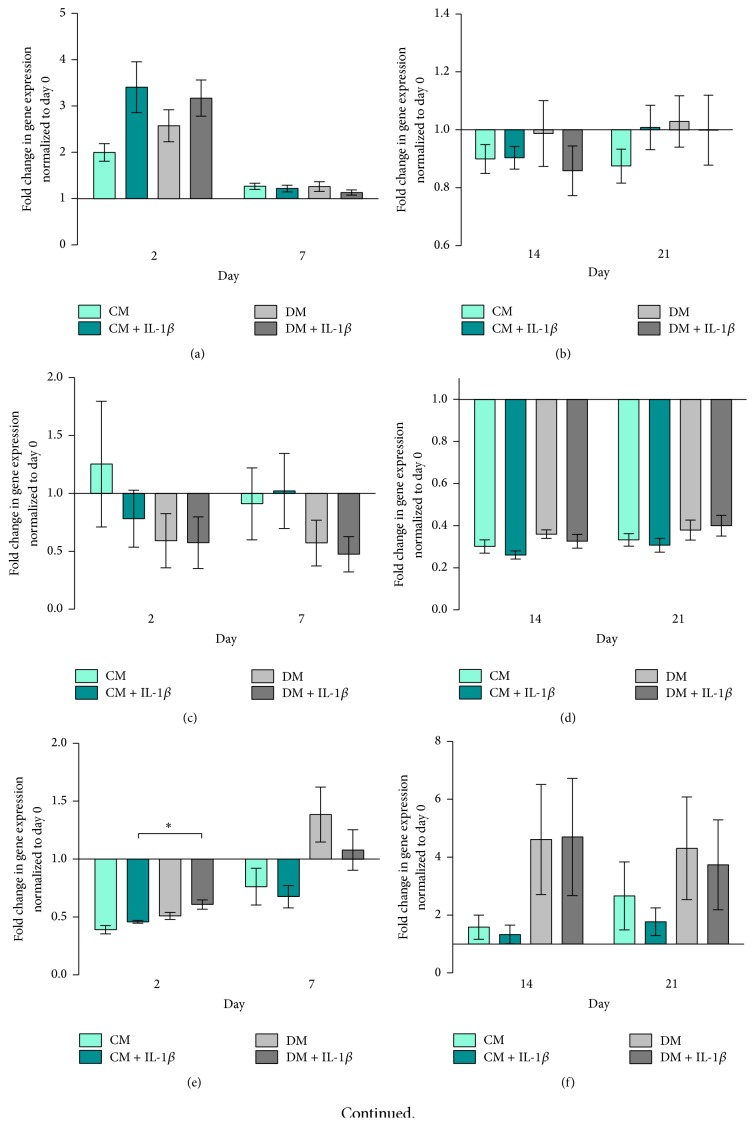
hMSC gene expression. Fold change in relative gene expression of hMSCs normalized to unstimulated hMSCs (RPLP at day 0) quantified by real-time PCR. Bars show the mean ± SEM of 3 different experiments performed in triplicate per group (*n* = 9 per group with the exception of (e) day 7 in CM + IL-1*β*, where *n* = 8). D'Agostino-Pearson omnibus normality test was used followed by one-way ANOVA,  ^*∗*^ *p* < 0.05. (a) Runx2 on days 2 and 7, (b) Runx2 on days 14 and 21, (c) Sox9 on days 2 and 7, (d) Sox9 on days 14 and 21, (e) ALP on days 2 and 7, (f) ALP on days 14 and 21, (g) Type 1 Col on days 2 and 7, (h) Type 1 Col on days 14 and 21, (i) OC on days 2 and 7, and (j) OC on days 14 and 21.

Runx2 expression was higher in IL-1*β* stimulated hMSCs, cultured in both CM and DM, compared to unstimulated cells on day 2 (Figure 2(a)). At day 7, expression of Runx2 fell and remained low throughout subsequent culture in all groups (Figures 2(a) and 2(b)). hMSCs cultured in differentiation medium showed slightly higher Runx2 gene expression levels on days 2 and 21 compared to control medium groups.

Sox9 expression was downregulated following osteogenic induction at days 2 and 7 (Figure 2(c)). IL-1*β* stimulation downregulated Sox9 expression in cells cultured under basal conditions at day 2 but not in cells cultured in DM (Figure 2(c)). At days 14 and 21, Sox9 was downregulated in all groups (Figure 2(d)).

ALP gene expression increased over time in all groups, with cells cultured in DM and DM + IL-1*β* exhibiting an earlier response compared to CM and CM + IL-1*β* groups (Figure 2(e)). On day 2, IL-1*β* stimulated cells cultured in DM showed significantly higher ALP expression compared to the CM + IL-1*β* group (*p* < 0.05, Figure 2(e)). ALP remained more highly expressed by cells cultured in DM compared to CM regardless of IL-1*β* stimulation at days 14 and 21 (Figure 2(f)).

With regard to Type I Collagen, hMSCs cultured in control medium (CM and CM + IL-1*β*) showed decreasing gene expression over time. In DM and DM + IL-1*β* groups, Type I Collagen gene expression levels peaked at day 14 and fell by day 21 to levels comparable to earlier time points (Figures 2(g) and 2(h)).

Osteocalcin (OC) gene expression was downregulated in all groups throughout the experiment (Figures 2(i) and 2(j)).

**Figure 3:**
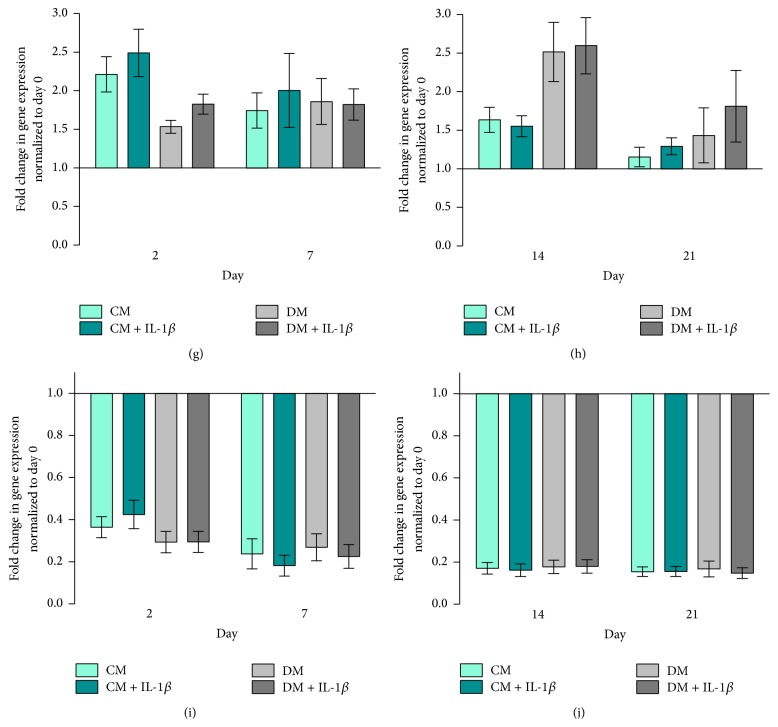
Runx2/Sox9 ratio hMSCs. Bars show the mean ± SEM of 3 different experiments performed in triplicate per group based on fold change in gene expression normalized to day 0 (*n* = 3 per group); one-way ANOVA was used to test for statistical significance. (a) Runx2/Sox9 ratio on days 2 and 7, (b) Runx2/Sox9 ratio on days 14 and 21 (mind the gap in the *y*-axis).

hMSCs cultured in DM ± IL-1*β* stimulation exhibited a slightly higher Runx2/Sox9 ratio compared to cells cultured in CM at days 2 and 7 (Figure 3(a)). Runx2/Sox9 ratios decreased at later time points with no significant differences between groups (Figure 3(b)).

### 3.2. Paracrine Modulation of MG63-GFP Gene Expression Is Dependent upon hMSC Differentiation Status

**Figure 4:**
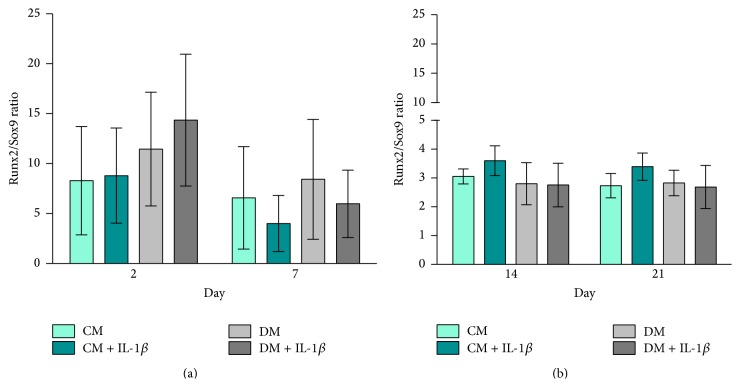
MG63-GFP gene expression. Fold change in relative gene expression of MG63-GFP cells normalized to RPLP at day 0 quantified by real-time PCR. Bars show the mean ± SEM of 3 different experiments performed in triplicate per group (*n* = 9 per group with the exception of (a, c, e, g, i, and k) in DM, where *n* = 8). Mean ± SEM; D'Agostino-Pearson omnibus normality test was used followed by one-way ANOVA;  ^*∗*^ *p* ≤ 0.05;  ^*∗∗*^ *p* ≤ 0.01;  ^*∗∗∗*^ *p* ≤ 0.001. (a) Runx2 on days 2 and 7, (b) Runx2 on days 14 and 21, (c) Sox9 on days 2 and 7, (d) Sox9 on days 14 and 21, (e) ALP on days 2 and 7, (f) ALP on days 14 and 21, (g) Type 1 Col on days 2 and 7, (h) Type 1 Col on days 14 and 21, (i) OC on days 2 and 7, and (j) OC on days 14 and 21.

MG63-GFP cells cocultured with IL-1*β* stimulated hMSCs upregulated Runx2 gene expression in both CM and DM media on day 2. Over time, Runx2 gene expression in all 4 groups decreased to comparable levels and then rose at day 21 (Figures 4(a) and 4(b)). MG63-GFP Runx2 gene expression showed a similar pattern to hMSCs, with high values on day 2, downregulation by day 7, and subsequent upregulation on day 21.

Sox9 expression was the highest in all groups on day 2 with downregulation on days 7–14 followed by upregulation on day 21 (Figures 4(c) and 4(d)). MG63-GFP cells cocultured in control medium exhibited significantly higher Sox9 expression compared to cells cocultured in differentiation medium at all time points (*p* ≤ 0.05, Figures 4(c) and 4(d)).

In all four MG63-GFP groups, ALP gene expression increased from day 2 to day 14 and then fell at day 21 (Figures 4(e) and 4(f)). ALP gene expression was significantly higher in CM and CM + IL-1*β* groups at later time points compared to cells cultured in differentiation medium ± IL-1*β* (Figures 4(e) and 4(f)).

Type 1 Collagen gene expression of MG63-GFP on day 2 was significantly higher in the CM + IL-1*β* compared to the CM group (*p* ≤ 0.05). Over time, CM groups showed a decrease in ALP expression (Figures 4(g) and 4(h)). MG63-GFP cells cocultured in DM displayed significant downregulation in ALP expression at all time points irrespective of IL-1*β* stimulation (Figures 4(g) and 4(h)).

Osteocalcin gene expression was consistently lower in DM groups compared to CM groups at all time points and was downregulated in all 4 groups at day 7 (Figure 4(i)). OC gene expression in all 4 groups increased again with maximum values at day 21 (Figure 4(j)). MG63-GFP cells in the DM + IL-1*β* group exhibited significantly lower OC gene expression on day 7 compared to cells in the CM + IL-1*β* group (*p* ≤ 0.01).

### 3.3. Elevated Runx2/Sox9 Ratio in MG63-GFP Cells Cocultured with IL-1*β* Stimulated hMSCs

**Figure 5:**
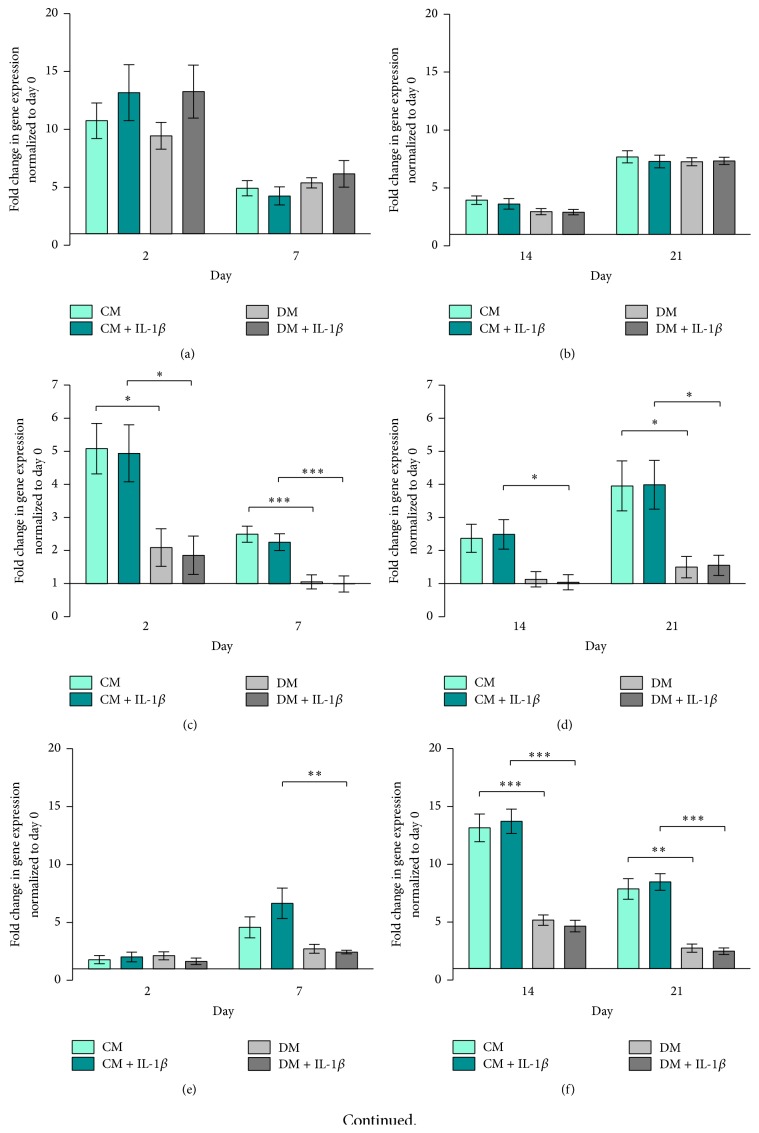
Runx2/Sox9 ratio MG63-GFP. Bars show the mean ± SEM of 3 different experiments performed in triplicate per group based on fold change in gene expression normalized to day 0 (*n* = 3 per group); one-way ANOVA was used to test for statistical significance,  ^*∗*^ *p* ≤ 0.05. (a) Runx2/Sox9 ratio on days 2 and 7, (b) Runx2/Sox9 ratio on days 14 and 21.

The Runx2/Sox9 ratio of MG63-GFP cocultured in CM ± IL-1*β* stimulation remained constant throughout the experiment. MG63-GFP cocultured in DM ± IL-1*β* stimulation presented consistently higher Runx2/Sox9 ratios compared to CM groups, which was significant at day 7 (DM + IL-1*β* versus CM + IL-1*β*, *p* < 0.05, Figures 5(a) and 5(b)).

### 3.4. Effect of Transient IL-1*β* Stimulation on Proliferation of hMSCs and MG63-GFP Cells

**Figure 6:**
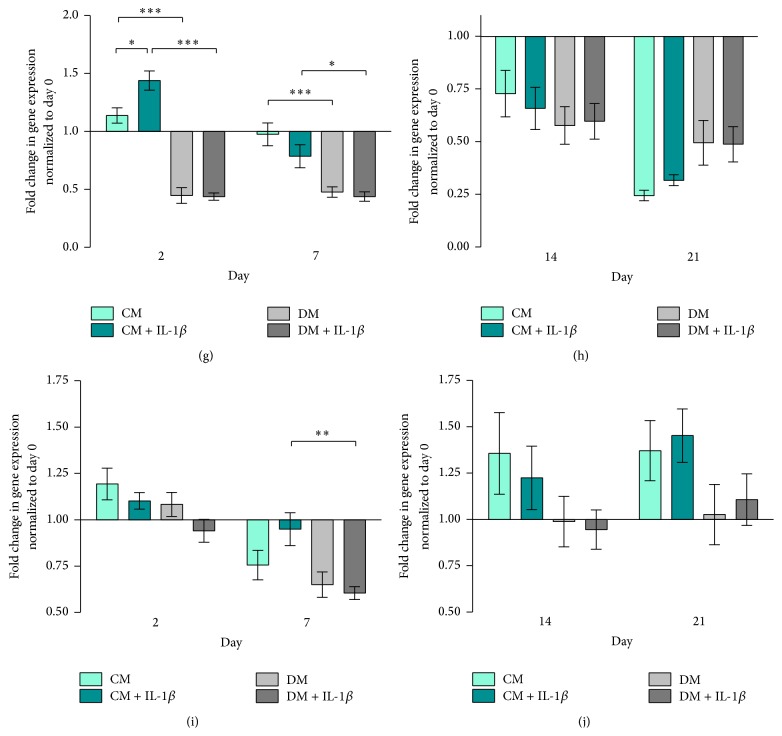
DNA quantification. Total DNA content expressed in *μ*g/mL. Bars show the mean ± SEM of 3 different experiments performed in triplicate per group. D'Agostino-Pearson omnibus normality test was used, followed by one-way ANOVA;  ^*∗*^ *p* ≤ 0.05;  ^*∗∗*^ *p* ≤ 0.01;  ^*∗∗∗*^ *p* ≤ 0.001. (a) Total DNA content in hMSC culture on days 14, 21, and 28 (*n* = 9 per group). (b) Total DNA content in MG63-GFP cell culture on days 14, 21, and 28 (*n* = 9 per group).

IL-1*β* stimulation had no effect on hMSC proliferation in coculture with MG63-GFP (Figure 6(a)). HMSCs cultured in DM showed significantly higher total DNA content compared to cells incubated in CM on the same day independent of IL-1*β* stimulation throughout the entire experiment, which is in line with published work [36]. Similarly, MG63-GFP cells showed significantly higher total DNA content in DM on all days analysed compared to cells cultured in CM. DNA content values for MG63-GFP cocultured in DM with unstimulated hMSCs (DM) were higher compared to cells cocultured with stimulated hMSCs (DM + IL-1*β*). This effect was detectable at all 3 time points with a significant difference on day 21 (*p* ≤ 0.05) (Figure 6(b)).

### 3.5. Transient IL-1*β* Stimulation Modulates ALP Activity in hMSCs and in MG63-GFP Cells in a Temporal Manner

**Figure 7:**
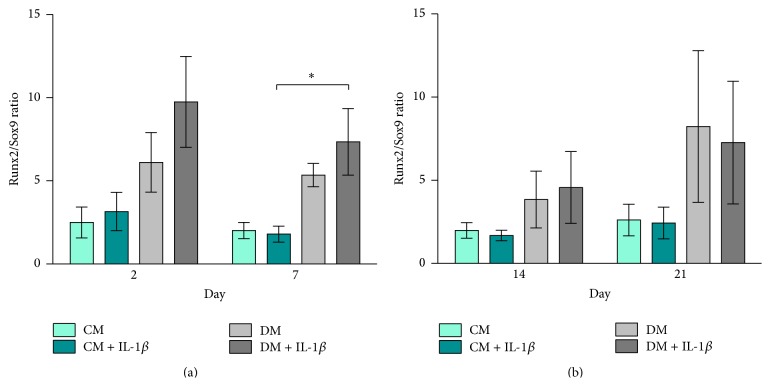
Alkaline phosphatase activity. ALP activity is related to total DNA content and expressed in nmol pNP/min/*μ*g DNA. Bars show the mean ± SEM of 3 different experiments performed in triplicate per group; one-way ANOVA was used to test for statistical significance;  ^*∗*^ *p* ≤ 0.05;  ^*∗∗*^ *p* ≤ 0.01. (a) ALP activity in hMSC culture on days 14 and 21 (*n* = 3 per group). (b) ALP activity in MG63-GFP cell culture on days 14 and 21 (*n* = 3 per group).

ALP activity related to total DNA content was increased in hMSCs that were stimulated and cultured in differentiation medium (DM + IL-1*β*) compared to unstimulated cells (DM) on days 14 and 21. Whilst, on day 14, stimulated hMSCs in control medium (CM + IL-1*β*) had slightly lower values compared to unstimulated cells (CM), on day 21, this correlation is reversed (Figure 7(a)).

ALP activity in MG63-GFP was significantly higher in MG63-GFP cells cultured in CM compared to cells cultured in DM on day 14 (*p* ≤ 0.05, *p* ≤ 0.01). Cells cultured with stimulated hMSCs showed higher ALP activity in both media (CM + IL-1*β* and DM + IL-1*β*) compared to coculture with unstimulated hMSCs. Increased ALP activity in MG63-GFP cells incubated with stimulated hMSCs cells was inverted on day 21 (Figure 7(b)). This effect indicates that IL-1*β* stimulation of hMSCs leads to an earlier response in ALP activity in MG63-GFP cells with higher values on day 14 and an earlier/stronger drop on day 21, which is in line with rt-PCR data where an earlier increase in gene expression is observed between days 2 and 7 (Figures 4(e) and 4(f)).

### 3.6. Transient IL-1*β* Stimulation Has Little Effect on Calcium Deposition in hMSCs Cocultured with MG63-GFP Cells

**Figure 8:**
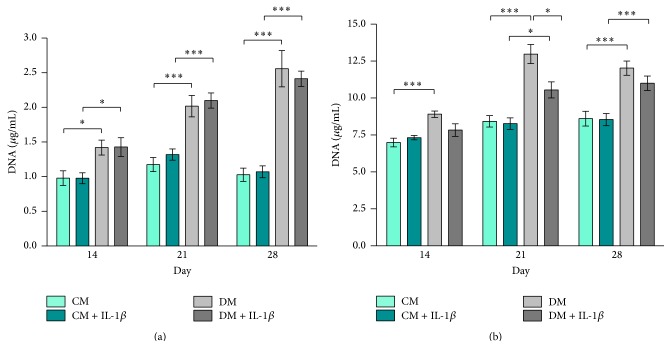
^45^Ca incorporation related to total DNA content. ^45^Ca incorporation is related to total DNA content and expressed in cpm/*μ*g DNA. Bars show the mean ± SEM of 3 different experiments performed in triplicate per group with the exception of (b) where 2 different experiments were performed; one-way ANOVA was used to test for statistical significance;  ^*∗∗*^ *p* ≤ 0.01;  ^*∗∗∗*^ *p* ≤ 0.001. (a) Incorporated ^45^Ca in hMSC culture on day 21 (*n* = 3 per group). (b) Incorporated ^45^Ca in MG63-GFP cell culture on day 21 (*n* = 3 per group).

HMSCs cultured in DM ± IL-1*β* showed significantly higher ^45^Ca incorporation on day 21 compared to controls (*p* ≤ 0.01). IL-1*β* stimulation of hMSCs resulted in slightly lower incorporation of the radioisotope ^45^Ca on day 21 in CM + IL-1*β* and DM + IL-1*β* groups compared to CM and DM groups (Figure 8(a)). Cocultured MG63-GFP showed similar results; however, none of the differences were significant. Culturing in DM resulted in overall significantly higher ^45^Ca incorporation on day 21 (*p* ≤ 0.01 and *p* ≤ 0.001). MG63-GFP cells cocultured with stimulated hMSCs in differentiation medium (DM + IL-1*β*) had lower incorporation numbers than cells cocultured with unstimulated hMSCs (DM) (Figure 8(b)).

**Figure 9:**
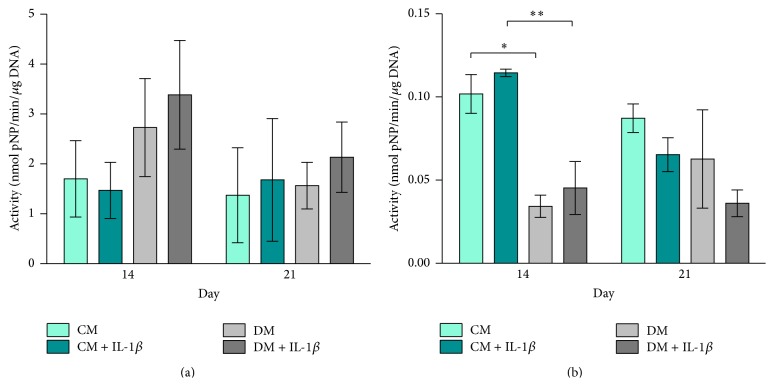
Alizarin Red S quantification. Bounded ARS is related to total DNA content and expressed in nmol/*μ*g DNA. Bars show the mean ± SEM of 3 different experiments performed in triplicate per group with the exception of (b) where 2 different experiments were performed; one-way ANOVA was used to test for statistical significance. (a) ARS content in hMSC culture on day 28 (*n* = 3 per group). (b) ARS content in MG63-GFP cell culture on day 28 (DM ± IL-1*β*,  *n* = 2 per group; CM ± IL-1*β*,  *n* = 3 per group). Notice: *y*-axis is gapped.

Stimulated hMSCs in CM, as well as in DM, contained slightly less bound ARS related to total DNA content than hMSCs without stimulation (Figure 9(a)). In line with the calcium data, these effects were not significant.

However, MG63-GFP cocultured with stimulated hMSCs showed similar levels of bound ARS when cultured either in control medium or in differentiation medium (Figure 9(b)).

**Figure 10:**
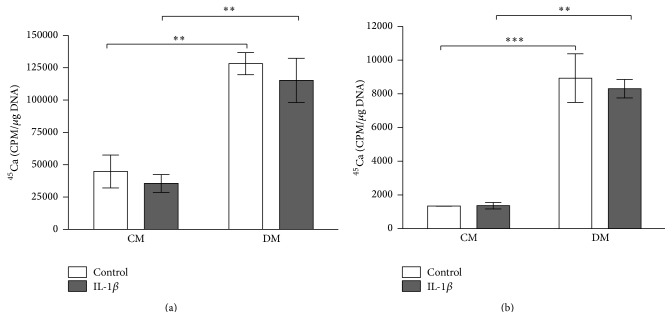
Alizarin Red S staining. Alizarin Red S staining of all groups. Results show representatives of 3 different experiments with similar findings. Row (a) (scale bar: 1 mm) and row (c) (CM and CM ± IL-1*β*, scale bar: 1 mm; DM and DM ± IL-1*β*, scale bar: 500 *μ*m) show 4x magnification. Rows (b) and (d) show 10x magnification (scale bar: 200 *μ*m).

Qualitative Alizarin Red S staining showed similar staining patterns comparing CM and CM + IL-1*β*, as well as DM and DM + IL-1*β* in both cell types. Culturing hMSCs in differentiation medium resulted in pronounced ARS staining independent of whether hMSCs were previously stimulated with IL-1*β* or not (Figure 10). Additionally, MG63-GFP (a cell line not normally associated with calcification) exhibited significantly greater ARS staining in DM versus CM (Figure 10).

Interestingly, low magnification images of MG63-GFP cells that had been cultured in differentiation medium (DM and DM + IL-1*β*) showed clear delineation between stained and nonstained areas (Figure 10(a)). Stained areas correlated with the size and form of the original micromass and were located directly under the membrane carrying the hMSCs. Even though cells were detected outside the staining border, these areas did not reveal calcium deposition.

## 4. Discussion

Proinflammatory cytokines released during the inflammatory response in the initial phase after a bone fracture exert a great influence on the behaviour of surrounding cells involved in the following healing process [10, 37]. IL-1*β*, as one of the most powerful proinflammatory cytokines, is upregulated in the early phase of bone healing after fracture and is fundamental in the regeneration cascade [17, 18, 38]. While so far many stimulation procedures are based on continuous administration [12, 20–22], it was previously shown that short-term stimulation of hMSCs resulted in a broad gene expression change that persisted over time [23, 24]. Differentiation of MSCs to an osteogenic phenotype has been shown to reduce the angiogenic profile of the cells secretome in both equine and human MSCs [39, 40]. However, the effect of MSC osteogenic differentiation on the cross talk with osteoblasts and their precursors is less clear. While continuous administrations protocols are costly and impractical in a direct clinical setting, short-term stimulation may be performed intraoperatively.

Here, we investigated the direct and indirect long-term effects of 2-hour short-term IL-1*β* stimulation of human MSCs cocultured with the osteosarcoma cell line MG63-GFP in a transwell system that allowed indirect cell communication.

### 4.1. IL-1*β* Stimulation of hMSCs Resulted in an Early mRNA Expression Response of Bone Markers

Following the 2-hour stimulation of hMSCs with interleukin-1*β*, we were able to demonstrate prompt downregulation of genes related to osteogenesis (Runx2, ALP, and Type 1 Col) with concomitant upregulation of Sox9 gene expression (Figure 1). However, by day 2, these effects were mostly reversed. These findings are in line with published results by Czekanska et al. who described significant downregulation of mRNA expression of the bone markers Runx2 and Sox9 48 hours after 2-hour stimulation [24]. In line with results published by Czekanska et al., we could detect a donor dependent variability in the Sox9 gene expression changes. In addition to changes at the mRNA level, Czekanska et al. investigated protein levels of cytokines in medium conditioned by stimulated hMSCs. Stimulation of hMSCs with IL-1*β* had a strong influence on protein synthesis, influencing the secretion of proteins involved in chemotaxis, inflammation, angiogenesis, and bone formation [24]. Based on these findings, we investigated the effect of stimulated hMSCs in an indirect coculture with cells of the osteoblast cell line MG63-GFP exhibiting an immature osteoblastic phenotype.

### 4.2. IL-1*β* Stimulation of hMSCs Resulted in Transient Changes in Gene Expression

Following temporary downregulation of osteogenic related genes 2 hours after stimulation of hMSCs with IL-1*β*, mRNA gene expression increased over time again. In line with previous published work, this effect was highly donor dependent but shows an overall similar pattern in gene expression [24].

Runx2, a key transcription factor involved in osteoblast differentiation and bone formation [41], is generally upregulated in the early phase upon stimulation of hMSCs. The gene expression of Sox9, a transcription factor related to chondrogenesis and endochondral bone formation [35], however is strongly downregulated. The ratio of Sox9 and Runx2 gene expression is considered to be a critical predictive factor of direct osteogenesis [41, 42]. We show here that IL-1*β* had a clear influence on the gene expression of both Runx2 and Sox9 at early time points. Not only did stimulated hMSCs show clear upregulation in the Runx2 gene expression in control medium (CM + IL-1*β*) on days 2 and 21 and differentiation medium (DM + IL-1*β*) at day 2, but also at the same time the stimulation of hMSCs resulted in strong downregulation of Sox9 gene expression. Similar results were presented by Murakami et al. in mouse chondrocytes [35] and by Wehling et al. in human MSCs [43]. The downregulation in Sox9 gene expression could also be detected in the unstimulated hMSC group cultured in differentiation medium (DM) and is also in line with previous published work [42].

The Runx2/Sox9 ratio was recently described as an important indicator for the responsiveness of human bone marrow derived hMSCs regarding their osteogenic differentiation potential [42]. By increasing the Runx2 expression and simultaneously decreasing Sox9 expression, IL-1*β* stimulated hMSCs showed a higher Runx2/Sox9 ratio in differentiation and culture medium in early time points with the highest ratio in DM + IL-1*β* group on day 2. While Loebel et al. attributed Runx2/Sox9 ratio on day 7 as a powerful predictor of osteogenic development, our data presented here confirm day 2 as being a useful time point as well [42]. Early changes induced by transient stimulation have been shown to have longer term effects. 30 min incubation of mouse MSCs with IGF-1 has been shown to increase cell survival after engraftment [44]. Further work would be required to establish the* in vivo* effect of transient IL-1*β* stimulation. At later time points, IL-1*β* stimulated cells cultured in control medium led to an even higher Runx2/Sox9 ratio than in cells cultured in differentiation medium. This would suggest that these cells were undergoing delayed spontaneous differentiation.

IL-1*β* stimulated hMSCs cultured in control medium demonstrated higher levels of Type 1 Collagen gene expression compared to unstimulated cells which peaked earlier in comparison to stimulated cells in differentiation medium. ALP gene expression is increased throughout the time and shows overall higher expression in cells cultured in differentiation medium.

Normally, Type 1 Collagen is upregulated during the proliferation phase, with its fibril formation being responsible for matrix formation* in vivo*, while ALP gene expression is upregulated during the early stages of matrix synthesis, peaking around day 14 and subsequently leading to matrix maturation and mineralisation [45].

The consistent downregulation of Osteocalcin in all 4 groups throughout the experiment is not unusual. Jaiswal et al. suggested that Osteocalcin might not be a reliable marker for osteogenic differentiation due to the inhibitory effect of dexamethasone on Osteocalcin mRNA expression [46].

Attention should be paid to the fact that the beneficial effect of IL-1*β* after a short administration time is mostly evident in early time points. However, this effect occurs during the critical initial engraftment period, when the healing response is initiated.

### 4.3. ALP Activity Is Enhanced after Stimulation without Changes in Calcium Deposition

Regarding the cell viability, neither IL-1*β* alone nor IL-1*β* in combination with dexamethasone reduced total DNA content in the hMSC samples, similar to previously published results [19]. Furthermore, continuous administration of IL-1*β* resulted in an increase of total DNA content [20]. In line with these results, Gowen et al. could show an increase in proliferation in human bone cells when cultured in IL-1 containing medium [47].

ALP is considered to be a marker for osteoblastic differentiation that reaches its maximum during early stages of matrix maturation [48]. Stimulation of hMSCs with IL-1*β* led to an increase in ALP activity. This effect was more obvious when cells were cultured in differentiation medium compared to the control group, which is in line with published work showing that cell cultures grown in the presence of dexamethasone presented induction of ALP activity [36, 49]. Ferreira et al. however did not detect any changes in ALP activity compared to cells cultured in medium containing dexamethasone. This might be explained by the long-term culturing/stimulation approaches with IL-1*β* [19].

Quantification of incorporated ^45^Ca revealed similar values in stimulated cell groups in both types of media. Comparable results could be found when looking at the ARS quantification.

In addition, it has to be taken into consideration that the Calcium-45 measurement only represents a sequence of the passing calcification/mineralisation process. This process is highly donor dependent and provides the information about the Calcium-45 uptake at that given time point. This can lead to misinterpretation, as an antedated calcification process would have lower uptake as an ongoing process.

Furthermore, qualitative comparison of ARS staining showed no clear difference between stimulated and unstimulated groups. This effect might be described by previous work claiming that it needed at least a minimum of 14 days of continuous IL-1*β* application to be effective in inducing mineralisation response [19]. These findings could be an explanation for the comparable values in the ^45^Ca incorporation and ARS quantification measurements. Similar results were described by Loebel et al. and Sonomoto et al. who administered the IL-1*β* for 10 and 28 days, respectively, in order to detect a distinct difference [20, 22].

### 4.4. Stimulated hMSCs Evoke Upregulation of Genes in MG63-GFP Cells

The stimulation of hMSCs and subsequent culturing in the two different media revealed diverse effects. While the stimulation of hMSCs in osteogenic medium depicted a strong stimulus towards an osteogenic differentiation behaviour in hMSCs, these cells subsequently lost their stimulatory capacities guiding MG63-GFP cells in their further differentiation at the mRNA level. Stimulated hMSCs cultured in control medium however abided in a more undifferentiated stage but exhibited a distinct capability to stimulate the cocultured MG63-GFP cells in their differentiation behaviour. MG63-GFP cells under the influence of stimulated hMSCs showed a stronger response in Type 1 Collagen gene expression on day 2 when cultured in control medium (CM + IL-1*β*) compared to the differentiation group (DM and DM + IL-1*β*). Over time, Type 1 Collagen gene expression was decreased and simultaneously the gene expression of ALP in MG63-GFP cells cocultured with stimulated hMSCs in control medium (CM + IL-1*β*) was increasingly upregulated. Starting around day 14, Osteocalcin gene expression in MG63-GFP cells cultured in control medium with un- or stimulated cells (CM and CM + IL-1*β*) started rapidly to increase. These effects were either not seen or just slightly distinct when cells were cocultured with hMSCs in differentiation medium (DM and DM + IL-1*β*). Concerning the observed gene expression pattern, differentiated hMSCs (upregulated ALP and Type 1 Col) exhibit a distinct impaired stimulatory effect towards MG63-GFP cells. Stimulated undifferentiated hMSCs (CM + IL-1*β*) instead take up a more regulatory/stimulatory role that evokes upregulation of gene expression in MG63-GFP cells.

It is noteworthy that the Type 1 Collagen, ALP, and OC gene expression in MG63-GFP cells cocultured with unstimulated hMSCs in differentiation medium (DM) were expressed to a higher level compared to previously published results [50].

Interestingly, MG63-GFP cells cultured in differentiation medium with stimulated hMSCs showed a significantly lower DNA content compared to the control group (DM) (Figure 6(b)). This effect was only visible when cells were cultured in differentiation medium.

Dedhar found regulation in the expression of the cell adhesion receptors (mainly integrins), stimulation of ALP activity, while cell proliferation was inhibited by direct stimulation of MG63 cells with recombinant human IL-1*β* [51]. Dedhar concluded that the stimulus would inhibit mainly cells that exhibit a high proliferation rate thereby shifting to a more differentiated osteoblastic cell type that expresses a higher alkaline activity [51]. Keeping in mind that only hMSCs were exposed to transient IL-1*β* stimulation, the changes in MG63-GFP phenotype indicate an indirect response to the hMSC conditioned environment. Moreover, Czekanska et al. detected upregulation of IL-1*β* gene expression 72 hours after stimulating hMSCs with IL-1*β* [24].

Czekanska et al. described in previous work a low ALP activity with a maximum of enzyme activity on day 28 in MG63 cultured as monolayer cell formation under the influence of osteogenic medium [50]. Interestingly, we detected the ALP activity increasing around day 14 with a decrease towards day 21, which is in line with the ALP gene expression pattern, with a maximum around day 14 in MG63-GFP cells cultured in control medium. Coculturing of MG63-GFP cells with stimulated hMSCs in micromass formation resulted in an earlier ALP activity peak in MG63-GFP cells compared to the other groups.

Striking are the opposite responses in ALP gene expression seen when comparing hMSCs and MG63-GFP cells. HMSCs cultured in differentiation medium show the highest ALP gene expression (Figures 2(e) and 2(f)), while MG63-GFP cells show strong upregulation of ALP gene expression when cocultured in control medium, whereas the cells cultured in differentiation medium feature a slight increase (Figures 4(e) and 4(f)). Besides the mentioned observation, ±IL-1*β* treatment reveals an additional remarkable effect: hMSCs treated with IL-1*β* (CM + IL-1*β*) have a contrary ALP expression behaviour in contrast to MG63-GFP cells in the coculture (CM + IL-1*β*). The same effect is detectable when comparing unstimulated hMSCs cultured in control medium (CM) and MG63-GFP (CM). Upregulation in ALP gene expression in hMSCs (±IL-1*β*) resulted in downregulation in MG63-GFP gene expression and vice versa.

Despite the dramatic changes seen in gene expression and ALP protein activity in MG63-GFP cells cocultured with hMSCs in control medium, no calcification was observed. This would suggest that a critical element is missing in MG63 at the later stages of differentiation, which is not sufficiently provided by the paracrine activity of naïve MSCs.

However, MG63-GFP cocultured with hMSCs in differentiation medium displayed robust calcification. Earlier studies have claimed a low protein synthesis in combination with a low ALP activity to be responsible for insufficient matrix maturation thereby leading to precluding calcification of MG63 cells [50]. Previous groups did detect a cell layer formed by MG63 cells after monolayer cell culture but without any signs of calcium deposition even after 28 days of cell culture [50, 52]. Here, we showed clear matrix mineralization in both groups cultured in differentiation medium (DM and DM + IL-1*β*) on day 28. This is the most novel aspect of the work presented; the mineralization of MG63 in coculture offers a unique system to investigate the critical steps of late stage osteogenic differentiation. One reason might be the different cell formation as we cultured the cells in a micromass construct. However, previous MG63-GFP micromass cultures did not calcify in the absence of hMSCs (data not shown). Considering the coculture with un- or stimulated hMSCs, it is also possible that by indirect cell communication MG63-GFP cells started to deposit calcium as the ARS stained only areas of the MG63-GFP cells formation that were directly located underneath the hMSCs cell formation. Considering the fact that the stained areas are located directly under the membrane carrying hMSCs, hMSCs seem to promote MG63-GFP calcification.

One possible explanation could be that secreted molecules by hMSCs fall due to gravity onto the MG63-GFP cells below the transwell system rather than being homogenously distributed in the medium. Thereby, only cells that are located directly underneath the membrane would be influenced by the secretome of hMSC cells. As soluble mediators would not exhibit such activity, a more likely explanation would be extracellular vesicles (EVs), containing proteins, mRNA, and microRNA, which could play a role as key elements of the stem cell paracrine activity [53, 54]. EVs include exosomes, which are 30–100 nm diameter vesicles of endocytic origin, and microvesicles (MVs), which are 100 nm–1 *μ*m diameter vesicles directly shed from the cell membrane [53, 54]. Vesicles produced by the osteogenically differentiated hMSCs would then be able to fall through the transwell membrane pores and thus signal to the cells directly below. The contents of these vesicles are the likely source of the required signals that allow the MG63-GFP cells to calcify.

## 5. Conclusion

Bone healing involves well-orchestrated interplay between cells and the surrounding cytokine milieu. Besides cell-cell contact, indirect cell interactions (by secreting a broad spectrum of growth and differentiation factors) play a major role in the ongoing healing process. Here, we show that short-term stimulation of hMSCs with IL-1*β* was sufficient enough to cause a change in gene expression. Besides having an impact on the differentiation process of immature hMSCs, less differentiated IL-1*β* stimulated hMSCs exhibit a more regulatory behaviour towards the surrounding cells, leading to upregulation of genes in MG63-GFP osteoblasts. These results indicate that short stimulation of hMSCs has the potential to modify short-term osteogenesis-related gene expression, as well as influencing the surrounding cell milieu, and offers a novel tool for clinical cell therapy approaches in translational medicine.

## Supplementary Material

Table 1: Corresponding primer and probe sequences of genes of interest used for real-time PCR.
