# Supplementary material for: Effect of Short-Term Stimulation with Interleukin-1β and Differentiation Medium on Human Mesenchymal Stromal Cell Paracrine Activity in Coculture with Osteoblasts
Source: Biomed Res Int. 2015 Dec 22;2015:714230. doi: 10.1155/2015/714230 (PMC4700155; doi:10.1155/2015/714230)
Supplement: Supplementary file 1 — Table 1: Corresponding primer and probe sequences of genes of interest used for real-time PCR. [file 714230.f1.pdf]

Supplemental Table

1 *Sequences used for PCR*

Gene	Forward 5′ – 3′	Reverse 3′ – 5′	Probe	Applied Biosystem reference number
**RPFL0**	5'-TGG GCA AGA ACA CCA TGA TG-3'	5'-CGG ATA TGA GGC AGC AGT TTC-3'	5'-AGG GCA CCT GGA AAA CAA CCC AGC-3'	
**Runx2**	5′-AGC AAG GTT CAA CGA TCT GAG AT-3′	5′-TTT GTG AAG ACG GTT ATG GTC AA-3′	5′-TGA AAC TCT TGC CTC GTC CAC TCC G-3′	
**Sox9**				Hs00165814_m1
**OC**	5′-AAG AGA CCC AGG CGC TAC CT-3′	5′-AAC TCG TCA CAG TCC GGA TTG-3′	5′- ATG GCT GGG AGC CCC AGT CCC-3′	
**ALPL**				Hs00758162_m1
**Col 1A1**	5′-CCC TGG AAA GAA TGG AGA TGA T-3′	5′-ACT GAA ACC TCT GTG TCC CTT CA-3′	5′-CGG GCA ATC CTC GAG CAC CCT-3′	
